# Erratum to “The structure of the Italian version of the Practice Environment Scale of the Nursing Work Index”

**DOI:** 10.1155/jonm/9837569

**Published:** 2025-08-07

M. Zanini, M. E. Musio, R. Watson, et al., “The structure of the Italian version of the Practice Environment Scale of the Nursing Work Index,” *Journal of Nursing Management* 30, no. 7 (2022): 3440–3448, https://doi.org/10.1111/jonm.13808.

In the article titled “The structure of the Italian version of the Practice Environment Scale of the Nursing Work Index,” there was an error in Section 3.4. This error is shown below:

The term “more likely” should read “less likely.”

The corrected section appears below:

**3.4 | Construct Validity**

Logistic regression showed that as the total value of Italian PES-NWI increased by one point, nurses were more than 10 times more satisfied with their work (OR = 10.33; 95% CI 8.62–12.35), and when the total value of Italian PES-NWI increased by one point, nurses were 14% less likely to want to leave their profession (OR = 0.14; 95% CI 0.12–0.16).

We apologize for this error.

